# The Cerefy^®^ Atlas of Cerebral Vasculature

**Published:** 2010-09-16

**Authors:** Justin Dye

**Affiliations:** Department of Neurosurgery, David Geffen School of Medicine, University of California, Los Angeles, CA, USA

**Figure:**
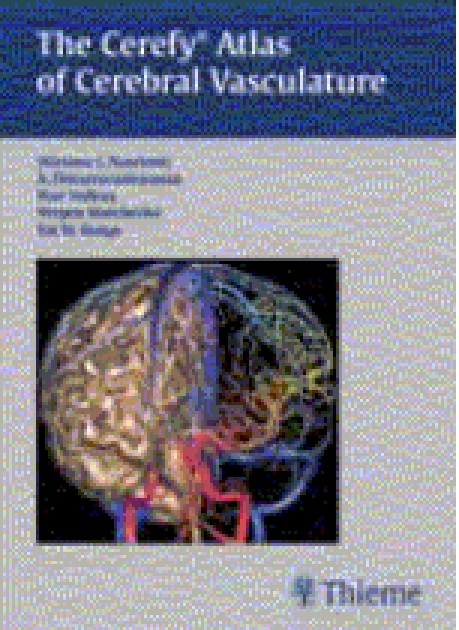


*The Cerefy^®^ Atlas of Cerebral Vasculature* is a detailed 3-dimensional (3D) atlas of cerebral arteries and veins. The current atlas was designed along the same principles that were used to create Thieme’s five previous brain atlases, namely *The Cerefy Atlas of Brain Anatomy*, published in 2006.

This interactive CD-ROM combines 3D drawings with magnetic resonance images (MRI) and magnetic resonance angiography (MRA) images. When first launching the exploration portion of the program, the 365 v-essels can be overwhelming when viewed all at once. However, it quickly becomes obvious that one of the strengths of this atlas is being able to select out one hemisphere of vessels, just arteries or just veins, one segment of the circle of Willis, or even a single distal MCA branch. What also sets this electronic atlas apart from more traditional textbooks is the fact that it allows the user to explore the vessels in 3D rather than the 2-dimensional (2D) page. The 3D cerebral vasculature model was created from a 3T MRA time of flight scan and was constructed manually by a vascular editor. The model is then co-registered with MRI and MRA scans from the same subject and displayed as a 3D triplanar image. In other words, the user can select 2D MRI or MRA images which can be scrolled through the 3D vasculature, providing a unique view of the relationship of the vessels to the surrounding parenchymal structures. These MRI/MRA scans can be viewed in axial, sagittal, or coronal planes. The user is also given the option of adding in drawings of the cerebral hemispheres and/or the ventricular system. This again adds to the 3D picture and reinforces the relationships of these vessels to the nearby parenchyma and ventricles in a way that most 2D atlases cannot.

It takes a short period of trial-and-error to learn the different functions available within the program. While there is no interactive tutorial available, there is a “user guide” and a “help” function. Once familiar with the different commands, it becomes easy to rotate the image to 360°, zoom in and out, and pan to the area of interest. Moving the cursor along the length of any vessel provides the user with the name and vessel diameter. Any relationship can also be quickly measured by dragging the cursor between two points of interest. Furthermore, by clicking anywhere along a vessel, the user is given the option of learning about its anatomy and variability, or seeing a list of references for further study. This is another major strength of this program. By selecting one of these options, a webpage automatically opens with a detailed description of the vessel of interest. Included in the CD-ROM are 100 images and 215 pages of text. There is also a test mode which is simple to use for review. There is even an option to save any image to your desktop for later review or presentations.

*The Cerefy^®^ Atlas of Cerebral Vasculature* is easy to use, unique, and a valuable learning tool for any student, resident, or practicing physician. What it adds to the current cerebral vascular textbooks is the ability to view the vessels “*in situ*” and gain a better understanding of their 3D relationships. I would strongly recommend this atlas for anyone interested in cerebral vasculature.

I give this atlas 4 out of 5 stars.

